# FUS Negatively Regulates Kaposi’s Sarcoma-Associated Herpesvirus Gene Expression

**DOI:** 10.3390/v10070359

**Published:** 2018-07-06

**Authors:** William Dunker, Yu Song, Yang Zhao, John Karijolich

**Affiliations:** 1 Department of Pathology, Microbiology, and Immunology, Vanderbilt University School of Medicine, Nashville, TN 37232, USA; william.dunker@vanderbilt.edu (W.D.); songyugzyx@163.com (Y.S.); yang.zhao.1@vanderbilt.edu (Y.Z.); 2 College of Pharmacy, Xinxiang Medical University, Xinxiang 453000, China; 3 Vanderbilt-Ingram Cancer Center, Vanderbilt University Medical Center, Nashville, TN 37232, USA; 4 Vanderbilt Institute for Infection, Immunology and Inflammation, Nashville, TN 37232, USA

**Keywords:** KSHV, RTA, viral lytic reactivation, FUS, RNA Polymerase II, Restriction Factor

## Abstract

Kaposi’s sarcoma-associated herpesvirus (KSHV) is a human gammaherpesvirus and the etiological agent of Kaposi’s sarcoma. KSHV is also causally associated with the development of lymphoproliferative diseases, including primary effusion lymphoma (PEL). KSHV reactivation from latency plays an integral role in the progression to KSHV-associated disease as several lytic proteins have angiogenic and anti-apoptotic functions essential to the tumor microenvironment. Thus, restriction of KSHV reactivation represents an attractive therapeutic target. Here, we demonstrate that the cellular protein Fused-in-sarcoma (FUS) restricts KSHV lytic reactivation in PEL and in an epithelial cell-based model. Depletion of FUS significantly enhances viral mRNA and protein expression, resulting in increased viral replication and production of infectious virions. Chromatin immunoprecipitation analyses demonstrate that FUS is present at several KSHV lytic cycle genes during the latent stage of infection. We further demonstrate that FUS interacts with RNA polymerase II and negatively affects Serine-2 phosphorylation of its C-terminal domain at the KSHV *RTA* gene, decreasing nascent RNA synthesis. Knockdown of FUS increases transcription of RTA, thus driving enhanced expression of KSHV lytic genes. Collectively, these data reveal a novel role for FUS in regulating viral gene expression and are the first to demonstrate its role as a viral restriction factor.

## 1. Introduction

Kaposi’s sarcoma-associated herpesvirus (KSHV) is a double-stranded DNA herpesvirus and belongs to the gammaherpesvirus subfamily. KSHV is an AIDS-associated opportunistic pathogen and the etiological agent of Kaposi’s sarcoma (KS), a multicentric, angioproliferative spindle cell tumor that is the most prevalent cancer in untreated AIDS patients [1,2,3,4]. The virus is also linked to various B cell lymphoproliferative diseases, including primary effusion lymphoma (PEL) and multicentric Castleman’s disease [5,6]. Similar to other herpesviruses, KSHV has two distinct phases of its lifecycle, latency and the lytic cycle. Latency is characterized by the persistence of an episome and expression of a limited number of viral genes [7]. In contrast, during viral reactivation, all viral genes are expressed, resulting in viral DNA replication and the production of infectious virions [8,9].

The latent to lytic transition is implicated in KSHV-associated disease progression, with both phases likely making unique contributions. For instance, latently expressed viral proteins have been shown to subvert cellular mechanisms which normally protect cells from aberrant proliferation, as is the case with latency-associated nuclear antigen (LANA), which can bind and inhibit tumor suppressors such as p53 and retinoblastoma protein [10,11]. Additionally, an anti-apoptotic transcriptional program is important for KSHV latency and the latent protein vFLIP has also been shown to activate the anti-apoptotic transcription factor NF-κB [12,13,14,15]. The importance of this gene expression program is highlighted by the essential nature of vFLIP for the survival of latent PEL cells [12]. While latent proteins have oncogenic properties, latency itself is not immortalizing, suggesting that lytic replication has a role. Consistent with this, clinical studies have shown the successful prevention as well as treatment of KSHV-associated malignancies with therapeutics that inhibit lytic replication [16,17,18,19,20]. It is now well appreciated that the presence of lytic cells is required for the production of angiogenic and anti-apoptotic viral products, which are essential to the tumor microenvironment [21,22,23].

The main driver of reactivation is the viral encoded replication and transcription activator (RTA/ORF50), which functions as a transcription factor and initiates expression of lytic cycle genes [24]. RTA is both necessary and sufficient to promote lytic gene expression [24,25]. Recombinant viruses that lack RTA, while capable of establishing latency, are unable to reactivate [26]. RTA is a sequence-specific transcriptional activator, consisting of an amino-terminal basic DNA-binding domain, a central leucine zipper motif, and a carboxy-terminal acidic activation domain [25]. RTA’s amino-terminal DNA-binding domain is capable of directly binding RTA-responsive elements within viral gene promoters with high affinity [27]. The amino-terminal domain and leucine zipper also promotes RTA’s interaction with cellular proteins, including Oct-1, RBP-Jκ, and K-RBP, that aid in viral promoter specification and transactivation by RTA [28,29,30,31,32]. The carboxy-terminal acidic activation domain is also required for RTA-mediated lytic reactivation and binds to cellular SWI/SNF and TRAP/Mediator complexes [33]. Despite low sequence homology within the carboxyl transcriptional activation domain among RTA homologs of gammaherpesviruses, the RTA, SWI/SNF, and TRAP/Mediator interactions are conserved in herpesvirus saimiri and murine gammaherpesvirus 68, highlighting the importance of cellular proteins in gammaherpesvirus reactivation [33].

Given RTA’s requirement for KSHV reactivation, it is perhaps not surprising that cellular proteins target RTA to prevent lytic replication. In fact, several proteins have been demonstrated to inhibit the transcriptional activation of RTA or limit RTA’s ability to transactivate lytic promoters. For instance, ADP-ribosylation and phosphorylation of RTA by PARP-1 and hKFC, respectively, result in the suppression of its recruitment to RTA-regulated lytic promoters [34]. Additionally, cellular KAP-1 associates with RTA-dependent lytic promoters and represses their expression, and depletion of KAP-1 is sufficient to induce KSHV reactivation [35].

It is likely other unidentified proteins affect RTA-mediated KSHV reactivation. Indeed, the human genome encodes numerous proteins that influence the dynamics of cellular transcription, and some have been tangentially associated with antiviral processes. For example, the cellular protein Fused-in-sarcoma (FUS) is involved in cellular transcription, and has also been demonstrated to be a component of cytoplasmic stress granules, which are important components of the host anti-viral response [36,37,38,39]. However, a role for FUS in the antiviral response has not been determined. Here, we demostrate FUS is a negative regulator of KSHV reactivation in PEL and in an epithelial cell-based model of KSHV infection. Loss of FUS leads to enhanced viral RNA and protein expression, resulting in an increase in the production of infectious virions. Chromatin immunoprecipitation (ChIP) analyses demonstrated that FUS is present at several KSHV loci, including RTA. Lytic reactivation promotes FUS eviction from many lytic cycle genes, however, FUS remains associated with *RTA*. Knockdown studies coupled with ChIP of RNA polymerase II (RNAP II) indicate that FUS negatively regulates phosphorylation on the C-terminal domain (CTD) of RNAP II at the RTA locus. Consistent with this, FUS knockdown increases RNAP II phosphorylation and increases nascent RNA expression of RTA, thus enhancing viral reactivation. These results demonstrate that FUS is an important cellular protein that negatively regulates viral gene expression and reveal a previously unknown antiviral role for FUS.

## 2. Materials and Methods

### 2.1. Cells and Viruses

iSLK.219 [40], iSLK.BAC16 [41], iSLK.Control [40], HEK293T (ATCC) were maintained in Dulbecco’s modified Eagle medium (DMEM; ThermoFisher, Waltham, MA, USA) supplemented with 10% fetal bovine serum (FBS; Invitrogen). TREx-BCBL1-RTA [42] were grown in RPMI 1640 medium (Invitrogen) supplemented with 10% fetal bovine serum (FBS; Invitrogen) and 2 mM l-glutamine (Invitrogen). All cells were maintained with 100 U of penicillin/mL and 100 μg of streptomycin/mL (Invitrogen) at 37 °C under 5% CO_2_. iSLK.219, iSLK.BAC16, iSLK.Control, and TREx-BCBL1-RTA cells were reactivated with 1 μg/mL of doxycycline (Fisher Scientific, Hampton, NH, USA).

### 2.2. Sirna Knockdowns

iSLK.219 cells were transfected at 60–80% confluency with 40 nM siRNA (FUS: 5′ rCrGrGrArCrAUrGrGrCrCUrCrArArArCrGrAdTdT) or MISSION siRNA Universal Negative Control #1 (Sigma, St. Louis, MO, USA) using Lipofectamine RNAiMax (Invitrogen). Forty-eight hours post-transfection, cells were reactivated as described above. The second FUS siRNA sequence is listed in Table S1. siRNAs were microporated into TREx-BCBL1-RTA cells using the Neon transfection system (Invitrogen) at 1600 v, 10 ms pulse width, and 3 pulses. Eighteen hours post-microporation, cells were reactivated as described above, with the addition of 100 μM sodium butyrate (Sigma).

### 2.3. Genome Replication

TREx-BCBL1-RTA cells were treated with FUS siRNA for 18 h, and reactivated with doxycycline and sodium butyrate for 48 h. The virus and cells were pelleted at 20,000 G for 2 h at 4 °C. Viral genomes were quantified by qPCR using serially diluted BAC16 as a standard curve.

### 2.4. Supernatant Transfer

iSLK.219 cells were reactivated with doxycycline for 72 h, after which the supernatant was collected. The supernatant was added to HEK293T cells with 8 μg/mL of polybrene (Sigma) and spun at 1000 rpm for 1 h at room temperature. Fresh media was then added and the cells were incubated for 48 h, followed by analysis.

### 2.5. Cloning, Lentiviral Production and Infection

FUS was PCR amplified from pENTR4-FLAG FUS (Addgene, Cambridge, MA, USA) and Gateway cloned into pLenti-CMV-tight-blast-dest vector (Addgene).

Lentivirus was prepared in HEK293T cells. Cells were transfected at 50–60% confluency with recombinant FUS, psPAX2 (lentiviral packaging), and pMD2.G (lentiviral envelope) (Addgene) using polyjet (SignaGen, Rockville, MD, USA). Seventy-two hours post-transfection, the supernatant was collected, mixed with 8 μg/mL of polybrene, and iSLK.219 cells were spinfected as described above. Cells were selected for 2 weeks in media containing 5 μg/mL blasticidin (Invivogen).

### 2.6. Nucleic Acid Isolation and Measurement

For analysis of gene expression by RT-qPCR, total RNA was isolated with TRIzol (Invitrogen) in accordance with the manufacturer’s instructions. RNA was DNase I (NEB) treated at 37 °C for 20 min and inactivated with EDTA at 70 °C for 10 min. cDNA was synthesized from DNase-treated RNA with random 9-mer (Integrated DNA Technologies) and M-MLV RT (Promega). qPCR was performed using the PowerUp SYBR Green qPCR kit (Thermo Scientific) with appropriate primers (Table S1).

### 2.7. 4sU. Labeling

iSLK.219 cells were treated with 500 μM 4sU (Abcam, Cambridge, UK) for 3 min prior to isolating RNA with TRIzol as described above. DNase-treated 4sU-containing RNA (50 μg) was incubated in biotinylation buffer (10 mM Tris [pH 7.4], 1 mM EDTA), and 5 μg MTSEA-biotin-XX (Biotium, Fremont, CA, USA) with constant rotation in the dark at room temperature for 2 h. RNA was then phenol-chloroform extracted and precipitated with isopropanol. The pellet was resuspended in nuclease-free water and mixed with 50 μL Dynabeads MyOne streptavidin C1 (Invitrogen) that had been pre-washed twice with nuclease-free water. Samples were rotated in the dark for 1 h at RT with high salt buffer (100 mM Tris [pH 7.5], 10 mM EDTA, 1 M NaCl, 0.1% Tween 20), then washed 4× with high salt wash buffer. Samples were eluted with fresh 5% β-mercaptoethanol, and the RNA was precipitated with ethanol prior to RT-qPCR.

### 2.8. Subcellular Fractionation and Western Blotting

Subcellular fractionation was performed using the REAP method with the minor modification of using one 10-cm plate for each fractionation condition [43].

Cell lysates were prepared with lysis buffer (50 mM Tris [pH 7.6], 150 mM NaCl, 0.5% NP-40) and quantified by Bradford assay. Equivalent amounts of each sample were resolved by SDS-PAGE, electrotransferred to PVDF membrane, and blotted for the indicated proteins. Antibodies: FUS (Santa Cruz; diluted 1:5000), GAPDH (Invitrogen; diluted 1:5000), H3 (Millipore, Burlington, MA, USA; diluted 1:1000), Hsp90 (Cell Signaling, Danvers, MA, USA; diluted 1:1000), RNAPII (Millipore; 1:1000), Ser2 RNAPII (Abcam; diluted 1:1000), RTA (diluted 1:10,000), ORF57 (diluted 1:1000), and bZIP (diluted 1:2000). Primary antibodies were followed by AlexaFluor 680-conjugated anti-rabbit and anti-mouse secondary antibodies (Life Technologies, Carlsbad, CA, USA; 1:5000) and visualized by Li-Cor Odyssey.

### 2.9. Immunoprecipitation (IP)

iSLK.219 cells were reactivated for the indicated time with doxycycline. Cells were washed in PBS, collected, and lysed in lysis buffer (50 mM Tris [pH 7.6], 150 mM NaCl, 0.5% NP-40, 10% glycerol). Three micrograms of RNAP II antibody (8WG16, Millipore) or control IgG (Cell Signaling) were incubated with SureBeads Protein G magnetic beads (Bio-Rad) at room temperature for 10 min, then added to the cell lysate. IPs were performed overnight at 4 °C with gentle rotation. Beads were washed three times in lysis buffer, followed by elution in 1X Laemmli buffer at 70 °C for 10 min.

### 2.10. Phosphonoacetic Acid (PAA) Treatment

iSLK.219 cells were transfected with FUS-siRNA (40 nM) at 60–70% confluency. Thirty-eight hours post-siRNA treatment, cells were treated with phosphonoacetic acid (PAA) (Alfa Aesar; final concentration: 0.5 mM). Four hours later, the cells were reactivated with doxycycline. Cells were harvested 24 and 48 h after induction, and viral gene expression was quantified by RT-qPCR.

### 2.11. Immunofluorescence Microscopy

iSLK.BAC16 cells, cultured on glass coverslips, were fixed in 4% paraformaldehyde (Ted Pella) for 15 min, permeabilized in ice cold methanol for 1 h, blocked in blocking buffer (1% Triton X-100, 0.5% Tween-20, 3% BSA, 5% normal goat serum (Invitrogen)) for 30 min, and incubated in primary antibody overnight (FUS: diluted 1:250). Slides were incubated with Rhodamine red anti-mouse secondary antibody (Thermo Scientific; diluted 1:750). Cells were imaged with an Olympus FV1000 confocal microscope.

### 2.12. Chromatin Immunoprecipitation (ChIP)

ChIP was performed as described previously [44], with the following modifications: chromatin was sheared by using a tip sonicator (Fisher Scientific) for 25 rounds of 20 s pulses with 20 Amplitude and 40 s off. Chromatin was diluted in ChIP dilution buffer (0.01% [*w*/*v*] SDS, 1.1% [*v*/*v*] Triton X-100, 1.2 mM EDTA, 16.7 mM Tris-HCl pH 8.1, 167 mM NaCl). ChIP was performed overnight at 4 °C using 5 μg RNAP II antibody (8WG16, Millipore), FUS (J2516, Santa Cruz), Ser2 RNAPII (5095, Abcam), with Control IgG (Cell Signaling) and rotated overnight at 4 °C. DNA was isolated after crosslink reversal using a QIAGEN PCR clean up kit prior to qPCR (Table S1).

## 3. Results

### 3.1. FUS Knockdown Increases KSHV Lytic Gene Expression and Virion Production

**Figure 1:**
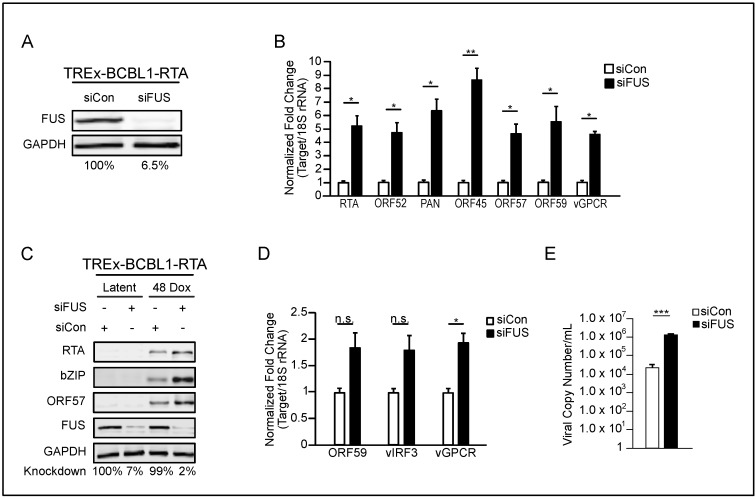
Fused-in-sarcoma (FUS) represses Kaposi’s sarcoma-associated herpesvirus (KSHV) reactivation in primary effusion lymphoma (PEL) cells. (**A**) Western blot analysis of FUS expression 48 h post reactivation in TREx-BCBL1-RTA cells treatmented with the indicated siRNAs. Knockdown efficiency is indicated beneath each lane. (**B**) RT-qPCR of viral transcripts from cells in (**A**). All samples were normalized to 18S and siCon levels set to 1. (**C**) Western blot of viral proteins from cells in (**A**). (**D**) TREx-BCBL1-RTA cells were transfected with the indicated siRNAs for 18 h prior to RNA isolation and RT-qPCR. All samples were normalized to 18S and siCon levels set to 1. (**E**) TREx-BCBL1-RTA cells were depleted of FUS for 18 h, followed by reactivation with doxycycline and sodium butyrate for 48 h. Virus and cells were centrifuged, viral genomes present were determined as described in the methods.. All samples were normalized to GAPDH, and siCon set to 1. Student *t* test used to determine statistical significance * *p* ≤ 0.05. ** *p* ≤ 0.005. *** *p* ≤ 0.0005, n.s. not significant.

FUS participates in the regulation of gene expression at multiple levels, including transcriptional regulation, and has also been implicated in B cell biology, the main latent reservoir of KSHV [36,37,38,45,46]. We hypothesized that given the extensive rewiring of gene expression during KSHV reactivation that FUS impacts the viral lytic phase. To test the role of FUS in KSHV B cell lytic reactivation we depleted FUS in TREx-BCBL1-RTA cells, a patient-derived PEL cell line harboring a doxycycline-inducible lytic transactivator RTA, using siRNA. Eighteen hours post-siRNA transfection, cells were reactivated with doxycycline and sodium butyrate for 48 h. FUS-targeted siRNA reduced its levels by 93.5% compared to the nontarget-siRNA (Figure 1A). Using RT-qPCR, we quantified the expression of KSHV genes representing different viral kinetic classes. FUS depletion resulted in a significant increase in the steady state levels of several KSHV-encoded mRNAs as well as the long noncoding RNA PAN (Figure 1B). Furthermore, we verified that protein levels of RTA, bZIP, and ORF57 were increased in FUS-depleted cells relative to control lytic cells (Figure 1C). A small percentage of PEL cells undergo spontaneous lytic reactivation at steady state. To investigate the role of FUS in spontaneous lytic reactivation of PEL cells, we depleted FUS in TREx-BCBL1-RTA cells with siRNA and quantified lytic transcript abundance by RT-qPCR. Depletion of FUS promoted a minor increase in expression of three lytic genes, presumably in the population of PEL cells undergoing spontaneous lytic reactivation (Figure 1D). Given the increase in viral lytic gene expression, we predicted that knockdown of FUS would affect virion production. Virus was harvested from the supernatant and cell pellets of induced TREx-BCBL1-RTA cells treated with control- or FUS-specific siRNA and quantified by qPCR. Indeed, knockdown of FUS resulted in an increase in viral production (Figure 1E).

**Figure 2:**
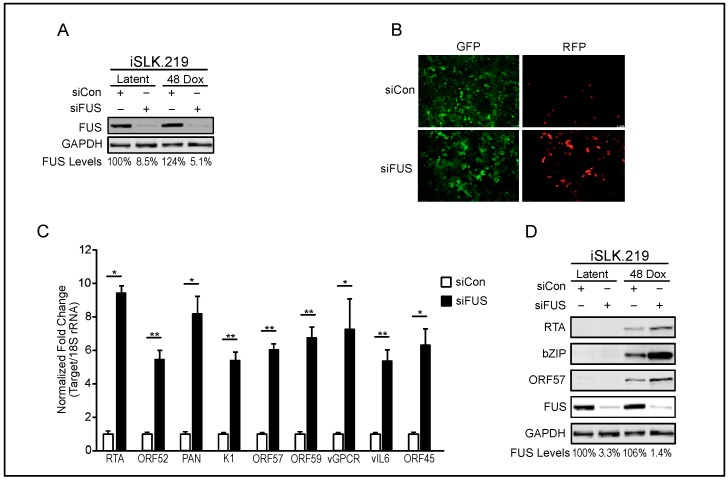
FUS represses KSHV reactivation in iSLK.219 cells. (**A**) iSLK.219 cells were treated with siRNA against FUS for 48 h, followed by doxycycline-induced reactivation of KSHV for 48 h. Knockdown efficiency in latency and 48 h post-reactivation was determined by western blot and is indicated beneath each lane. GAPDH was used as a loading control. (**B**) Fluorescent microscopy, at 10× magnification, of iSLK.219 cells depleted of FUS by siRNA in (**A**). (**C**) RT-qPCR of viral transcripts from FUS-depleted iSLK.219 cells in (**A**). All samples were normalized to 18S and siCon levels set to 1. (**D**) Western blot of viral proteins from cells in (**A**). GAPDH was used as a loading control. Student *t* test used to determine statistical significance * *p* ≤ 0.05. ** *p* ≤ 0.005.

We also investigated the role of FUS in iSLK.219 cells. iSLKs are a clear-cell renal-cell carcinoma cell line (SLK) that stably maintains the KSHV.219 episome (iSLK.219) [40,47]. The recombinant KSHV.219 virus constitutively expresses green fluorescent protein (GFP) from the EF-1 alpha promoter and can be used as a proxy for the presence of KSHV within cells. The KSHV.219 virus also encodes red fluorescent protein (RFP) under the control of a viral lytic promoter. Similar to TREx-BCBL1-RTA cells, iSLK cells contain a doxycycline-inducible version of the lytic transactivator RTA. siRNA-depletion of FUS in iSLK.219 cells during latency and 48 h post-reactivation was efficient, reducing FUS levels by 91.5% and 94.9%, respectively (Figure 2A). Forty-eight hours post-reactivation, GFP and RFP positive cells were analyzed by fluorescence microscopy in FUS- and control-siRNA-treated cells. FUS depletion resulted in a marked increase in the number of RFP positive cells, suggesting more efficient entry into the lytic cycle (Figure 2B). Using RT-qPCR we quantified the expression of nine lytic genes. All viral transcripts analyzed were expressed greater than 4-fold more in FUS-depleted cells relative to control-siRNA treated cells (Figure 2C). Additionally, protein levels of RTA, bZIP, and ORF57 were increased in FUS-depleted lytic iSLK.219 cells relative to control-siRNA treated cells (Figure 2D).

**Figure 3:**
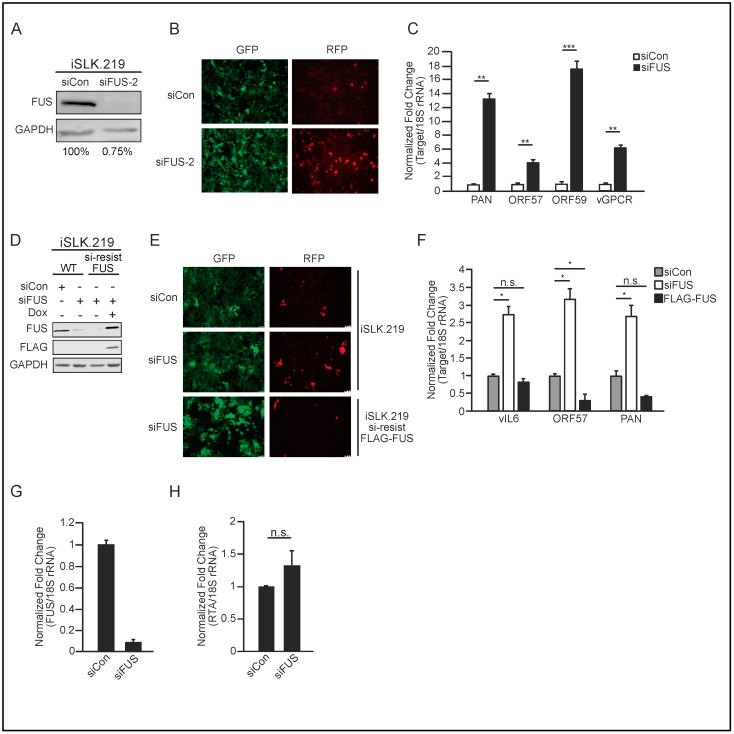
Independent verification of FUS-siRNA specificity. (**A**) iSLK.219 cells were depleted of FUS with a second FUS-siRNA and induced with doxycycline for 48 h. Knockdown efficiency was determined by western blot analysis and is indicated beneath each lane. (**B**) Fluorescent microscopy, at 10× magnification, of iSLK.219 cells depleted of FUS in (**A**). (**C**) Quantification of viral gene expression in cells described in (**A**) by RT-qPCR. All samples were normalized to 18S and siCon set to 1. (**D**) Western blot analyses of protein extracts from WT iSLK.219 and iSLK.219 cells transduced with siRNA-resistant FLAG-tagged FUS. Cells were treated with either control or FUS-siRNA and induced for 48 h with doxycycline. (**E**) Fluorescent microscopy, at 10× magnification, of cells in (**D**). (**F**) RT-qPCR of viral gene expression in cells described in (**D**). All samples were normalized to 18S and siCon set to 1. (**G**) Uninfected iSLK control cells, which harbor the doxycycline-inducible RTA, were depleted of FUS with siRNA for 48 h, followed by doxycycline treatment for 24 h. FUS knockdown was determined by RT-qPCR. (**H**) Quantification of RTA mRNA levels from cells in (**G**). Student *t* test used to determine statistical significance * *p* ≤ 0.05. ** *p* ≤ 0.005. *** *p* ≤ 0.0005, n.s. not significant.

To test whether the observed FUS phenotype was due to off-target effects of siRNA-mediated knockdown, we examined the effect of a second FUS-siRNA on KSHV reactivation. This second FUS- siRNA was highly effective, depleting levels of FUS to 0.75% relative to control-siRNA treated cells (Figure 3A). Forty-eight hours post-reactivation, cells depleted with the second FUS-siRNA exhibited greater RFP fluorescence than cells treated with control siRNA, suggesting more efficient lytic reactivation upon FUS depletion (Figure 3B). Indeed, RT-qPCR quantification of viral gene expression revealed increased PAN, ORF57, ORF59, and vGPCR expression in FUS-depleted cells relative to control siRNA treatment (Figure 3C).

To further demonstrate specificity of the FUS siRNA, we used lentiviral transduction to establish iSLK.219 cells harboring doxycycline-inducible, FLAG-tagged siRNA-resistant FUS, and quantified the effect of FUS siRNA treatment on KSHV lytic reactivation. Western blot analysis demonstrated that while endogenous FUS was significantly depleted by FUS-siRNA, siRNA-resistant FLAG-tagged FUS was not (Figure 3D). Importantly, expression of siRNA-resistant FUS in iSLK.219 cells abrogated the increased viral reactivation and gene expression observed in WT iSLK.219 cells treated with siRNA targeting FUS (Figure 3E,F).

The RT-qPCR primers to quantify RTA expression are specific to KSHV encoded RTA, however, given that both TREx-BCBL1-RTA and iSLK.219 cells are reactivated by doxycycline-induced expression of RTA, we verified that FUS depletion did not influence the expression of the doxycycline-inducible RTA. To investigate this, we treated iSLK cells that do not contain KSHV with control or FUS-specific siRNA and quantified their effect on doxycycline-induced RTA expression. With 91% depletion of FUS, we did not observe a significant change in RTA expression (Figure 3G,H).

**Figure 4:**
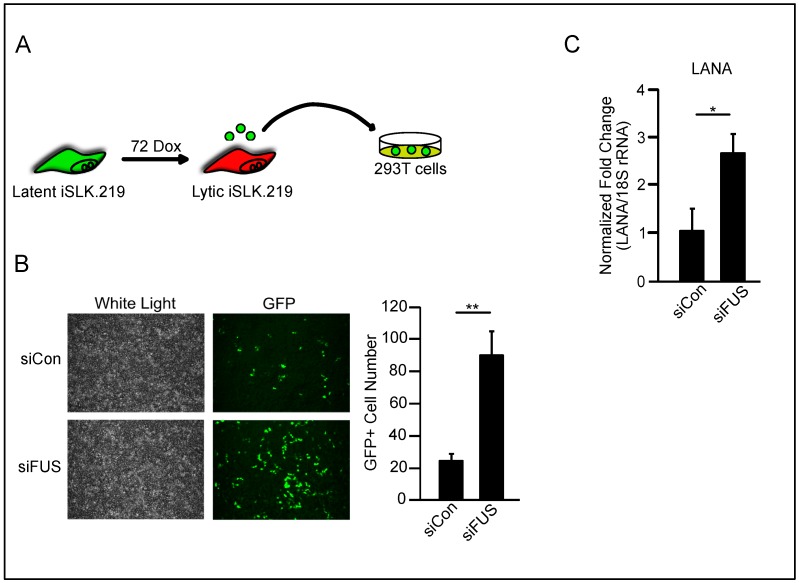
FUS restricts KSHV virion production in iSLK.219 cells. (**A**) Schematic of supernatant transfer. (**B**) Fluorescent microscopy, at 10× magnification, of KSHV-infected HEK293T cells following supernatant transfer from FUS-depleted iSLK.219. GFP-positive cells were infected with the virus. Quantification of GFP-positive cells is to the right. Values represent four independent views of the infected cells. (**C**) LANA mRNA levels from HEK293T cells in (**B**). Student *t* test used to determine statistical significance * *p* ≤ 0.05. ** *p* ≤ 0.005.

Having confirmed the specificity of the FUS-siRNA and the effect of its depletion on lytic gene expression, we next leveraged the fact that the KSHV.219 virus constitutively expresses GFP and quantified infectious virion production using a supernatant transfer assay. Following FUS- or control-siRNA treatment, iSLK.219 cells were reactivated for 72 h, whereupon supernatants were collected and used to infect HEK293T cells (Figure 4A). Infection of HEK293T cells using supernatants from iSLK.219 cells treated with FUS-specific siRNA resulted in a 4-fold increase in GFP-positive cells compared to supernatants from control-siRNA treated cells (Figure 4B). Additionally, we quantified the expression of the latent viral transcript LANA by RT-qPCR. Consistent with the number of GFP-positive cells, significantly more LANA was expressed in HEK293T cells infected with virions from FUS-siRNA treated iSLK.219 cells compared to control-siRNA treated cells (Figure 4C). Collectively, these results establish that FUS restricts KSHV reactivation and production of infectious virions in both PEL and iSLK.219 cells. This is the first demonstration of an antiviral role for FUS.

### 3.2. FUS Depletion Enhances KSHV Gene Expression Prior to Viral DNA Replication

**Figure 5:**
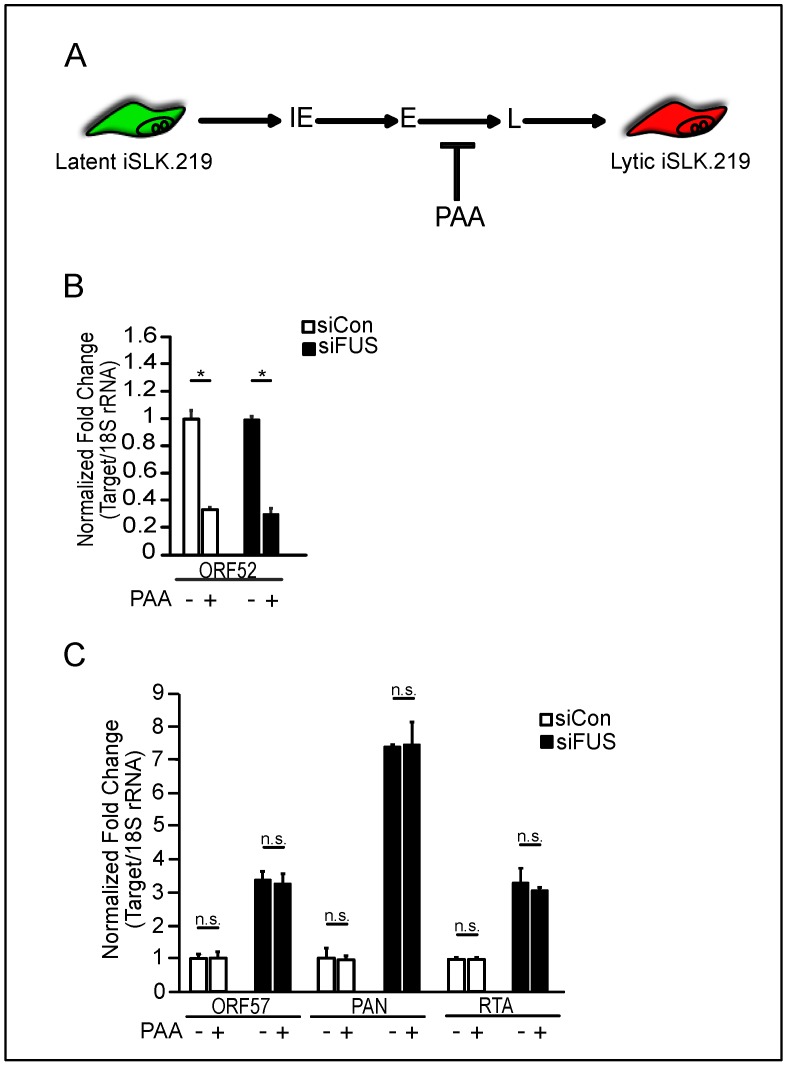
FUS restricts viral reactivation prior to DNA replication. (**A**) Schematic of the herpesviral gene expression cascade. Phosphonoacetic acid (PAA) treatment blocks viral DNA replication, thereby blocking late viral gene expression. IE, immediate early; E, early; L, late (**B**) Total RNA isolated from iSLK.219 cells reactivated for 48 h and transfected with either siCon or siFUS and treated with PAA, was subjected to RT-qPCR to monitor expression of ORF52. All samples were normalized to 18S. The non-PAA treated samples for both siCon and siFUS were normalized to 1 and compared to the PAA treated samples. (**C**) RT-qPCR of ORF57, PAN, and RTA viral genes from iSLK.219 cells treated with same conditions as (**B**) and collected at 24 h post reactivation. All samples were normalized to 18S. For each condition and gene, siCon was set to 1. Student *t* test used to determine statistical significance * *p* < 0.05, n.s. not significant.

We next sought to determine at which stage of lytic reactivation FUS affects KSHV gene expression. Herpesvirus gene expression occurs in a transcriptional cascade, wherein immediate-early genes are first transcribed, followed by early gene expression and viral DNA replication, and then late gene expression (Figure 5A). To determine where in the transcriptional cascade FUS affects viral gene expression, FUS- and control-siRNA treated iSLK.219 cells were treated with the viral DNA polymerase inhibitor phosphonoacetic acid (PAA), and viral early and late gene expression was quantified following reactivation. PAA treatment resulted in a significant decrease in the expression of the KSHV late gene *ORF52*, consistent with the dependency of late gene expression on viral replication (Figure 5A,B). Viral gene expression was significantly increased in FUS-siRNA treated cells compared to control-siRNA treated cells, and PAA treatment did not reduce the effect of FUS-siRNA (Figure 5C). These results indicate FUS restricts KSHV reactivation early during reactivation, prior to viral DNA replication and independent of late gene expression.

### 3.3. FUS is Nuclear Localized throughout KSHV Viral Reactivation

**Figure 6:**
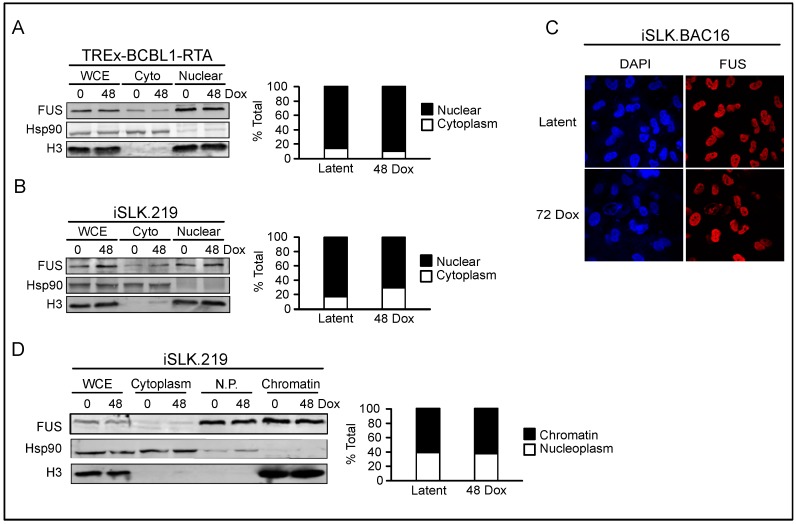
FUS is predominately nuclear in KSHV-infected cells. (**A**) TREx-BCBL1-RTA cells were fractionated at latency and 48 h post-reactivation. WCE: Whole Cell Extract. Cyto: Cytoplasm. Hsp90 is a cytoplasmic control, H3 is a nuclear control. Quantification is to the right of the western blot. (**B**) Fractionation of iSLK.219 cells. (**C**) IF, at 60× magnification, of iSLK.BAC16 cells at latency and 72 h post-reactivation. DAPI is a nuclear marker. (**D**) Chromatin fractionation of iSLK.219 cells at latency and 48 h post-reactivation. WCE: whole cell extract. N.P.: nucleoplasm. Hsp90 is a cytoplasmic control, H3 a nuclear control. Quantification is to the right of the Western blot.

The early effect of FUS on KSHV reactivation led us to hypothesize that FUS regulates the early transcriptional dynamics on the KSHV genome. However, FUS is a nucleocytoplasmic shuttling protein and can reside in either the cytoplasm or nucleus depending on the cellular state, and stress has been shown to promote its cytoplasmic relocalization [39,48]. Thus, we monitored FUS localization in TREx-BCBL1-RTA cells during latency and lytic reactivation by subcellular fractionation. Western blot analyses demonstrated that FUS predominantly localized to the nucleus in both latency and 48 h post-lytic reactivation (Figure 6A).

We also monitored FUS localization in iSLK.219 cells in both latency and lytic reactivation. Similar to TREx-BCBL1-RTA cells, FUS was predominately nuclear, and no cytoplasmic redistribution was detected upon lytic reactivation (Figure 6B). FUS localization was also determined in iSLK.BAC16 cells by immunofluorescence (IF) microscopy, which confirmed our biochemical fractionation data indicating a nuclear residence (Figure 6C). These cells are similar to iSLK.219 cells, however they do not express RFP upon lytic reactivation. Within the nucleus FUS has been demonstrated to associate with chromatin, thus we further fractionated the iSLK.219 nuclear fractions into soluble nucleoplasm and chromatin-bound fractions [36,37,38,46]. Western blot analyses demonstrated that approximately 60% of FUS fractionated with chromatin in both latency and the lytic cycle (Figure 6D). Collectively, these results demonstrate that FUS is nuclear localized in KSHV infected cells and its subcellular localization is not altered upon lytic reactivation.

### 3.4. FUS Affects RNA Polymerase II CTD Phosphorylation and Nascent RNA Transcription

**Figure 7:**
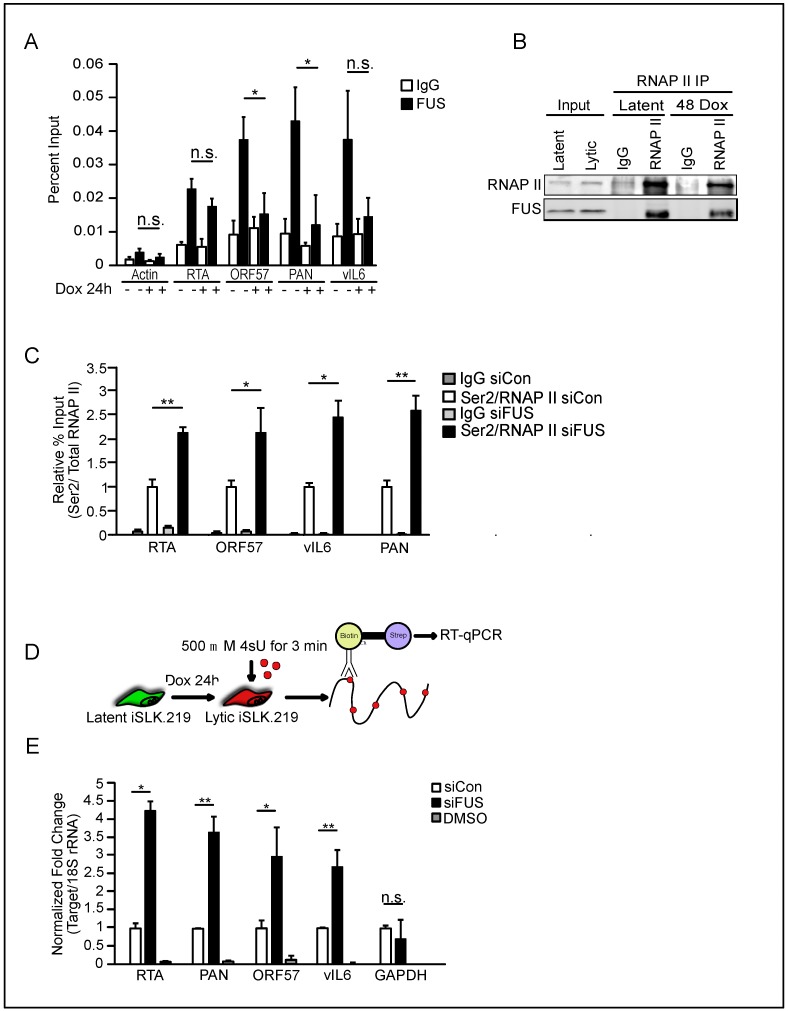
FUS regulates RTA nascent RNA expression. (**A**) FUS occupancy of the indicated viral genes was analyzed by Chromatin immunoprecipitation (ChIP) in iSLK.219 cells 24 h post-reactivation. IgG was used as a negative control. (**B**) Western blot of RNAP II immunoprecipitation (IP) from iSLK.219 cells at latency and 48 h post-reactivation. (**C**) Ser2 RNAP II occupancy was determined at the indicated viral genes by ChIP 48 h post-reactivation in iSLK.219 cells treated with either siCon or siFUS. Ser2 levels were normalized to total RNAP II present at each genomic loci as determined by ChIP. siCon ratio set at 1. (**D**) Schematic representation of the assay employed to measure nascent RNA synthesis. Cells are labeled with 500 mM 4sU for 3 min prior to total RNA isolation. Labeled RNA is subjected to thiol-specific biotinylation, followed by streptavidin purification and RT-qPCR. (**E**) RT-qPCR of nascent viral and host transcripts. Values were normalized to 18S. DMSO was added in place of 4sU as a negative control. Student *t* test used to determine statistical significance * *p* ≤ 0.05. ** *p* ≤ 0.005, n.s. not significant.

Prompted by the presence of FUS in the chromatin fraction, we tested whether FUS is present on the viral genome. We performed ChIP on FUS in both latent and 48 h post-reactivation lytic iSLK.219 cells and quantified its levels at select viral genes. FUS was present at several loci, including *RTA*, *ORF57*, *PAN*, and *vIL6* in latent cells (Figure 7A). Interestingly, during lytic reactivation, the levels of FUS decreased at *ORF57* and *PAN*, while quantitatively it was not significantly altered at the *RTA* and *vIL6* loci. However, the decrease at *vIL6* was trending towards significance.

RNAP II transcription is regulated by a dynamic cycle of post-translational modifications, including phosphorylation of the C-terminal domain (CTD) [49]. Interestingly, FUS has been demonstrated to interact with RNAP II and prevent Serine (Ser)2 CTD phosphorylation, a mark associated with elongation, at select cellular genes [36,37]. The recruitment of FUS to the *RTA* locus prior to reactivation, coupled with its maintenance during reactivation, could allow FUS to interact with RNAP II and influence Ser2 phosphorylation and thus RTA expression. To test this hypothesis, we first examined the interaction between FUS and RNAP II in latent and reactivated iSLK.219 cells by immunoprecipitation and western blotting. We observed a robust interaction in nuclear extracts between FUS and RNAP II, confirming that this interaction can occur in the context of KSHV latency and lytic reactivation (Figure 7B). Next, we monitored the presence of Ser2 phosphorylated RNAP II on the viral genome by ChIP. The levels of Ser2 phosphorylated RNAP II were normalized to the total levels of RNAP II. We observed that Ser2 phosphorylation was increased at the *RTA* locus, as well as at *ORF57*, *PAN*, and *vIL6* in FUS-siRNA treated cells relative to control-siRNA treated cells (Figure 7C). Furthermore, consistent with the ChIP data, we observed a significant increase in nascent RNA synthesis of RTA, PAN, ORF57, and vIL6 in FUS-depleted cells relative to control-siRNA treated cells by 4-thiouridine (4sU) pulse labeling and purification coupled to RT-qPCR (Figure 7D,E). Quantification of the housekeeping gene GAPDH from purified 4sU-labeled RNA showed no change in nascent RNA synthesis (Figure 7E). Collectively, these results are consistent with a mechanism whereby FUS recruitment and maintenance at the RTA locus reduces its expression, thus restricting KSHV reactivation.

## 4. Discussion

Reactivation from latency is necessary for the continual seeding of new infected cells, transmission of the virus to other individuals, and progression to KSHV-associated disease. As such, preventing or limiting lytic reactivation represents an attractive target for cellular restriction factors. Here, we demonstrate that the cellular protein FUS limits KSHV lytic reactivation in PEL and iSLK.219 cells. Although stress can promote nucleocytoplasmic shuttling of FUS, in the context of lytic reactivation, FUS remains predominately nuclear with a significant portion associated with chromatin [39,48]. FUS has previously been shown to interact with RNAP II and regulate CTD phosphorylation, and our immunoprecipitation and ChIP analyses suggest that FUS interacts with RNAP II and reduces Ser2 CTD phosphorylation at the RTA locus [36,37]. Consistent with FUS negatively affecting Ser2 CTD phosphorylation, siRNA-mediated knockdown of FUS robustly stimulates RTA nascent RNA production. These data put forth a model whereby FUS regulates RNAP II elongation at the RTA locus, and thus inhibits expression of the KSHV major lytic transactivator. Our study is the first to demonstrate an antiviral role for FUS and establish that it imposes a restriction on KSHV reactivation at the level of viral gene transcription.

We and others have demonstrated that nuclear FUS is able to regulate gene transcription [36,46,50]. While recruitment of FUS to the genome can occur through interactions with the CTD of RNAP II, how FUS is recruited to the viral episome during latency is unclear. However, FUS does have the ability to directly bind nucleic acids, including both single-stranded and double-stranded DNA and RNA, and these interactions could facilitate FUS occupancy on the KSHV genome [51]. If FUS recruitment was mediated via RNA, this could occur through either cis- or trans mechanisms. In cis, overlapping transcription of latent and lytic genes could provide the necessary RNAs, while in trans the RNA can be derived from either the cellular or viral genome. Investigations in to how FUS is recruited to viral DNA are underway and should reveal novel insight into its newly described antiviral function.

Previously, work from Schwartz et al. identified a role for FUS in regulating Ser2 CTD phosphorylation [36]. Ser2 phosphorylation can be catalyzed by multiple kinases, including the positive transcription elongation factor b (P-TEFb), CDK12, and dual-specificity tyrosine-regulated kinase (DYRK1A), and in vitro FUS directly inhibits the ability of P-TEFb to phosphorylate GST-CTD [36,52,53,54]. The mechanism by which FUS limits Ser2 CTD phosphorylation on the viral genome has not been elucidated. However, P-TEFb has been demonstrated to be present on the viral genome, and thus FUS inhibition of Ser2 CTD phosphorylation at viral genes may occur through P-TEFb inhibition, as proposed for select cellular genes [55]. Interestingly, Tsai et al. demonstrated that RTA is a target of CDK9, a kinase within the P-TEFb complex, and this phosphorylation is important for RTA-mediated transactivation [56]. Whether FUS also affects RTA-mediated transactivation via preventing P-TEFb mediated phosphorylation of RTA is worth pursuing.

FUS is an important regulator of B cell development and activation, and considering B cells are the main reservoir of KSHV latency, it will be interesting to determine how KSHV infection impacts the role of FUS in these processes [45]. Furthermore, mutations in FUS are associated with a range of diseases including cancer and amyotrophic lateral sclerosis (ALS). In the case of ALS, FUS mutations have been described that promote its relocalization to the cytoplasm [57]. Our work demonstrates that FUS restricts KSHV lytic reactivation within the nucleus at the level of viral transcription. Thus, we would predict KSHV reactivation would be significantly enhanced within cells harboring FUS mutations associated with ALS.

Beyond KSHV, our study raises the question of whether FUS restricts other viral pathogens. Epstein-Barr virus (EBV) is a closely related gammaherpesvirus and shares a similar tropism and lifecycle to KSHV. Whether FUS interacts with the latent EBV episome to regulate gene expression will be interesting to investigate. Similarly, other members of the Herpesviridae family establish latency as an episome. It is intriguing to speculate that FUS may function as a general restriction factor for this class of viruses. Collectively, this work has identified the cellular protein FUS as a novel KSHV restriction factor negatively regulating viral gene transcription.

## Supplementary Materials

The following are available online at http://www.mdpi.com/1999-4915/10/7/359/s1, Table S1: qPCR primers used in this study and the second FUS-specific siRNA sequence.
